# Improving antibiotic use through behaviour change: a systematic review of interventions evaluated in low- and middle-income countries

**DOI:** 10.1093/heapol/czab021

**Published:** 2021-04-05

**Authors:** Carla Cuevas, Neha Batura, Luh Putu Lila Wulandari, Mishal Khan, Virginia Wiseman

**Affiliations:** Centre for Global Health Economics, Institute for Global Health, University College London, 30 Guilford Street, London WC1N 1EH, UK; Centre for Global Health Economics, Institute for Global Health, University College London, 30 Guilford Street, London WC1N 1EH, UK; The Kirby Institute, University of New South Wales, Level 6, Wallace Wurth BuildingHigh Street, UNSW Australia. Sydney, New South Wales, 2052, Australia; Department of Global Health and Development, Faculty of Public Health and Policy, London School of Hygiene and Tropical Medicine, 15-17 Tavistock Place, London, WC1H 9SH, UK; Department of Global Health and Development, Faculty of Public Health and Policy, London School of Hygiene and Tropical Medicine, 15-17 Tavistock Place, London, WC1H 9SH, UK; Aga Khan University, National Stadium Road, Karachi, Pakistan; The Kirby Institute, University of New South Wales, Level 6, Wallace Wurth BuildingHigh Street, UNSW Australia. Sydney, New South Wales, 2052, Australia; Department of Global Health and Development, Faculty of Public Health and Policy, London School of Hygiene and Tropical Medicine, 15-17 Tavistock Place, London, WC1H 9SH, UK

**Keywords:** Antibiotic use, behaviour change, systematic review, LMICs

## Abstract

Antibiotic resistance (ABR) has been identified as a critical threat to global health at the highest policy fora. A leading cause of ABR is the inappropriate use of antibiotics by both patients and healthcare providers. Although countries around the world have committed to developing and implementing national action plans to tackle ABR, there is a considerable gap in evidence about effective behaviour change interventions addressing inappropriate use of antibiotics in low- and middle-income countries (LMICs), where ABR is growing at an alarming rate. We conducted a systematic review to synthesize evidence about the effectiveness and cost-effectiveness of behaviour change interventions to reduce inappropriate use of antibiotics in LMICs. Three databases were searched using a set of predefined search terms and exclusion criteria. The search identified 43 relevant articles. A narrative synthesis of results was conducted using the Behaviour Change Wheel framework to categorize intervention components. The majority of the reviewed studies were set in lower-middle-income or low-income countries located in Sub-Saharan Africa or East Asia and the Pacific. Twenty-four articles evaluated multi-faceted interventions over a period of 12 months or less. Despite the widespread use of antibiotics in the community, interventions were primarily implemented in public health facilities, targeting health professionals such as doctors, nurses, and other allied medical staff. Although education for providers was the most widely used strategy for influencing antibiotic use, it was shown to be most effective when used in conjunction with training or other enabling and supportive measures to nudge behaviour. Six articles included an evaluation of costs of interventions and found a reduction in costs in inpatient and outpatient settings, and one article found a training and guidelines implementation-based intervention to be highly cost-effective. However, the small number of articles conducting an economic evaluation highlights the need for such analyses to be conducted more frequently to support priority setting in resource-constrained environments.

**KEY MESSAGES**

- Behaviour change interventions that used education-based strategies either as a stand-alone intervention or as part of a multi-faceted intervention showed a positive impact on the use of antibiotics, compared to other strategies such as training, enablement or persuasion.
- The majority of studies evaluated interventions that targeted the behaviour of healthcare providers in public health facilities, and only a few focused on patients and the wider community or pharmacy staff, particularly in the private sector.
- The evidence base for effective interventions in low-income countries was weak and is likely to hinder the development of national action plans to curb antibiotic resistance.
- There is a dearth of evidence on which interventions are cost-effective and affordable, which can limit the ability of a decision-maker to gauge the relative value of investment in interventions that have the potential to address antibiotic resistance.

## Introduction

Antibiotic resistance (ABR) threatens our ability to cure common infectious diseases such as pneumonia, tuberculosis and gonorrhoea. It often results in prolonged illnesses as patients tend to remain infectious for longer periods of time (Neu, 1992), in turn increasing the risk of resistant infections spreading to other individuals (Mølbak, 2005; Holmes *et al.*, 2016). ABR also leads to the use of alternative and often more expensive and lengthy treatment procedures that place a considerable economic strain on individuals, their families and communities (Holmberg *et al.*, 1987; Paladino *et al.*, 2002; Mølbak, 2005; Holmes *et al.*, 2016) as well on resource-constrained healthcare systems (Okeke *et al.*, 2005; Arnold and Straus, 2009; Espinoza Franco *et al.*, 2009). Thus, it comes as no surprise that ABR has emerged as a growing threat to public health and societal well-being (Sumpradit *et al.*, 2012; Llor and Bjerrum, 2014). ABR is also a political and financial priority (Khan *et al.*, 2019) (Hernando-Amado *et al.*, 2019), as reflected in the global health agenda of the United Nations General Assembly in September 2016 (World Health Organization (WHO), 2016a); and the Global Health Security Agenda and the International Health Regulations (World Bank, 2017). Along with this, an estimated US$40 billion has also been mobilized to fund strategies to address ABR (O’Neill, 2016).

The inappropriate prescription, dispensing, consumption and use of antibiotics (henceforth, antibiotic use) in clinical settings by providers (including primary care, hospitals and private drug sellers) and by patients has been identified as a key driver of ABR (Espinoza Franco *et al.*, 2009; World Health Organization (WHO), 2016b). Inappropriate use of antibiotics includes, but is not limited to, treatment of conditions for which antibiotics are not clinically warranted, suboptimal dosage regimens, premature cessation of antibiotic treatment, lack of or poor quality consultation with healthcare providers, purchasing antibiotics without prescription and sharing antibiotics with others (Levy-Hara *et al.*, 2011; Smith *et al.*, 2018; Atif *et al.*, 2019). A complex range of factors determine the inappropriate use of antibiotics in LMICs. Studies report on a variety of supply-side factors such as a lack of knowledge among prescribers or habitual prescribing that is not in line with best practice (Radyowijati and Haak, 2003; Espinoza Franco *et al.*, 2009; Esmaily *et al.*, 2010; Holloway, 2011; Ayukekbong *et al.*, 2017; Suy *et al.*, 2019), inadequate medical education, training and supervision (Wahlstrom *et al.*, 2003; Holloway, 2011; Xiao *et al.*, 2013; Liang *et al.*, 2014); pharmaceutical promotion (Radyowijati and Haak, 2003; Holloway, 2011; Liang *et al.*, 2014; Yip *et al.*, 2014); inadequate interaction times between health workers and patients (Holloway, 2011; Llor and Bjerrum, 2014); inaccurate perceptions of patient needs and demands (Radyowijati and Haak, 2003; Holloway, 2011; Liu *et al.*, 2016); limited availability of diagnostic support tools (Holloway, 2011; Llor and Bjerrum, 2014); and the inappropriate prescription of drugs (Radyowijati and Haak, 2003; Holloway, 2011; Aiken *et al.*, 2013).

Studies also identify demand-side factors that relate to how individuals use or consume prescribed medicines. Commonly observed patient behaviour includes the overuse of antibiotics over unnecessarily long periods (Chan *et al.*, 2012); non-adherence to appropriate or recommended treatment (Radyowijati and Haak, 2003; Ayukekbong *et al.*, 2017) or the non-indicated use of antibiotics for uncomplicated viral infections such as upper respiratory tract infections (Owens *et al.*, 2004; Chan *et al.*, 2012). The reasons for these behaviours are varied and do not act independently of each other. They include high expectations or beliefs of how effective antibiotic treatment could be (Balabanova *et al.*, 2009); poor availability of information and lack of knowledge about the appropriate use of drugs for different conditions (Holloway, 2011; Ayukekbong *et al.*, 2017); the ability to easily access medicines over the counter without a prescription (Espinoza Franco *et al.*, 2009); and a strong culture or norm of self-prescribing medicines (Balabanova *et al.*, 2009). Geographical or economic barriers to accessing health facilities where prescription-based medicines may be obtained are also widely reported in the literature (Pavin *et al.*, 2003; Suy *et al.*, 2019).

Any strategy that aims to curb the spread of ABR must tackle these multi-dimensional supply- and demand-side behaviours in clinical care and community settings (World Health Organization (WHO), 2018) and extend to animal health and commercially driven agricultural settings, where the inappropriate use of antibiotics can further exacerbate ABR (Laxminarayan *et al.*, 2013). This is likely to involve several stakeholders such as government and non-governmental organizations, civil society, the private sector and academic institutions working across public health, animal health and the environment (One Health Platform, no date). This presents a challenge to policy formulation due to the competing priorities and diverse solutions offered by these different stakeholders (Khan *et al.*, 2019). Strategies to effectively address the public health threat posed by ABR would ideally need to achieve two goals: one, ensure access to effective treatment for common infections; and two, reduce the risk of emergence of ABR (Bloom *et al.*, 2017).

Five systematic reviews have identified interventions designed to improve antibiotic and antimicrobial stewardship (Arnold and Straus, 2009; Charani *et al.*, 2011; Davey *et al.*, 2013, 2017; Cross *et al.*, 2017; Wilkinson *et al.*, 2018). Three of these reviews included a handful of interventions implemented in LMICs (Charani *et al.*, 2011; Davey *et al.*, 2017; Wilkinson *et al.*, 2018). Davey *et al.*’s (2017) review focused on interventions to improve antibiotic use in inpatient settings and included five studies in LMICs. Charani *et al.* (2011), reviewed interventions to improve AB use among at clinicians and the general public but focused quite narrowly on mass media or campaign based and reviewed one study in an LMIC. The review by Wilkinson *et al.* (2018) focused on supply-side interventions in the public and private sector to improve antibiotic prescription in LMICs but did not include demand side interventions. No formal or established framework was used to categorize intervention strategy characteristics in this review, though some behaviour change interventions were identified. None of the five reviews analysed the cost and cost effectiveness of interventions, which is important as it provides policymakers with evidence on the relative value of investment in health intervention and aids efficient and equitable resource allocation (Drummond *et al.*, 2005; Guinness and Wiseman, 2011; Vassall *et al.*, 2017). This leaves a considerable knowledge gap for behaviour change with respect to the use of antibiotics in LMICs where ABR is growing rapidly and resources may be constrained (Yip *et al.*, 2014; Ayukekbong *et al.*, 2017; Wilkinson *et al.*, 2018). This limited evidence is likely to inhibit progress on the development of effective national ABR mitigation strategies (World Health Organisation, 2018).

Our review aims to fill this evidence gap by summarizing and critically appraising the literature on the effectiveness and cost-effectiveness of behaviour change interventions implemented in LMICs to improve the use of antibiotics in the domain of human health. This will be achieved by:

- Identifying behaviour change interventions for improved use of antibiotics in inpatient, outpatient and community settings in LMICs;
- Synthesizing available evidence on the effectiveness and cost-effectiveness of behaviour change interventions, using a published framework for behaviour change;
- Appraising the quality of the studies included in the review using the GRADE checklist (Atkins *et al.*, 2004);
- Discussing intervention types that are most strongly associated with effectiveness and cost-effectiveness; and
- Identifying knowledge gaps that can help inform future research and policy agendas on ABR in LMICs.

## Methods

Details of the methodology used for this review have been published in a review protocol (Batura *et al.*, 2018). A summary of the review methods is presented below.

### Search strategy

Two researchers independently conducted comprehensive searches for peer-reviewed articles using three research databases: Web of Science, PubMed and Google Scholar. This was followed by a hand search of the references included in the final set of papers to capture any additional papers that met the inclusion criteria (see below). The search terms are presented in Table 1.

**Table 1:** Keywords for systematic review search

Population—drugs	antibiotic*; antimicrobial*; ‘anti-bacterial agents’; antibacterial; anti-bacterial
Interventions	‘behavioural intervention*’, ‘behavioral intervention*’, ‘behaviour intervention’, ‘behavior intervention’, ‘behaviour change’, ‘behavior change’, ‘behaviour modification’, ‘behavior modification’, ‘training’, ‘supervision’, ‘education’, ‘knowledge’, ‘feedback’, ‘audit’, ‘reminders’, ‘modelling’, ‘modeling’, ‘enablement’, ‘persuasion’, ‘incentivisation’, ‘incentivization’, ‘coercion’, ‘restriction’, ‘environmental restructuring’, ‘guidelines’, ‘stewardship’, ‘law enforcement’, ‘policy’, ‘governance’
Outcomes	‘use’, ‘rational use’, ‘irrational use’, ‘inappropriate use’, ‘appropriate use’, ‘appropriate treatment’, ‘treatment’, ‘prescription’, ‘adequate prescription’, ‘prescri*’, ‘knowledge’, ‘prophylactic use’, ‘prophilaxys’, ‘effectiveness’, ‘cost effectiveness’, ‘cost-effectiveness’, ‘economic evaluation’, ‘costs’, ‘costing’, ‘cost effectiveness analysis’, ‘cost-effectiveness analysis’, ‘cost benefit analysis’, ‘cost-benefit analysis’, ‘cost utility analysis’, ‘cost-utility analysis’, ‘utilization’, ‘utilisation’, ‘drug use’, ‘medicine use’, ‘essential medicine*’, ‘drug information’, ‘drug therapy’, ‘consumption’, ‘prescribing practices’, ‘prescribing behaviour’, ‘prescribing behavior’
Countries	‘low and middle income countr*’, ‘low income countr*’, ‘middle income countr*’, LMIC*, ‘developing countr*’, Afghanistan, Benin, Burkina Faso, Burundi, Central African Republic, Chad, Comoros, Democratic Republic of Congo, Eritrea, Ethiopia, The Gambia, Guinea, Guinea Bissau, Guinea-Bissau, Haiti, Democratic People's Republic of Korea,, Liberia, Madagascar, Malawi, Mali, Mozambique, Nepal, Niger, Republic of Yemen, Yemen, Rwanda, Senegal, Sierra Leone, Syria, Somalia, South Sudan,, Tajikistan, Tanzania, Togo, Uganda, Zimbabwe, Angola, Argentina, Bangladesh, Bhutan, Bolivia, Cabo Verde, Cambodia, Cameroon, Republic of Congo, Congo, Cote d'Ivoire, Djibouti, Arab Republic of Egypt, Egypt, El Salvador, Georgia, Ghana, Honduras, India, Indonesia, Kenya, Kiribati, Kosovo, Republic of Kyrgyz, Kyrgyz, Lao PDR, Lao, Lesotho, Mauritania, Federated States of Micronesia, Micronesia, Moldova, Mongolia, Morocco, Myanmar, Burma, Nicaragua, Nigeria, Pakistan, Papua New Guinea, Philippines, Sao Tome and Principe, Solomon Islands, Sri Lanka, Sudan, Swaziland, Arab Republic of Syria, Timor-Leste, Timor Leste, East Timor, Tunisia, Ukraine, Uzbekistan, Vanuatu, Vietnam, West Bank and Gaza, Zambia, Albania, Algeria, American Samoa, Armenia, Azerbaijan, Belarus, Belize, Bosnia and Herzegovina, Botswana, Brazil, Bulgaria, China, Colombia, Costa Rica, Cuba, Dominica, Dominican Republic, Equatorial Guinea, Guinea, Ecuador, Fiji, Gabon, Grenada, Guatemala, Guyana, Islamic Republic of Iran, Iran, Iraq, Jamaica, Jordan, Kazakhstan, Lebanon, Libya, Republic of Macedonia, Macedonia, Malaysia, Maldives, Marshall Islands, Mauritius, Mexico, Montenegro, Namibia, Nauru, Paraguay, Peru, Romania, Russian Federation, Russia, Samoa, Serbia, South Africa, St Lucia, St Vincent and the Grenadines, Suriname, Thailand, Tonga, Turkey, Turkmenistan, Tuvalu, Venezuela RB, Venezuela
Terms within each row are separated by OR Terms across each row are separated by AND Limited to publications related to Humans Limited to publications since January 1990 to 2019	

**Figure 1:**
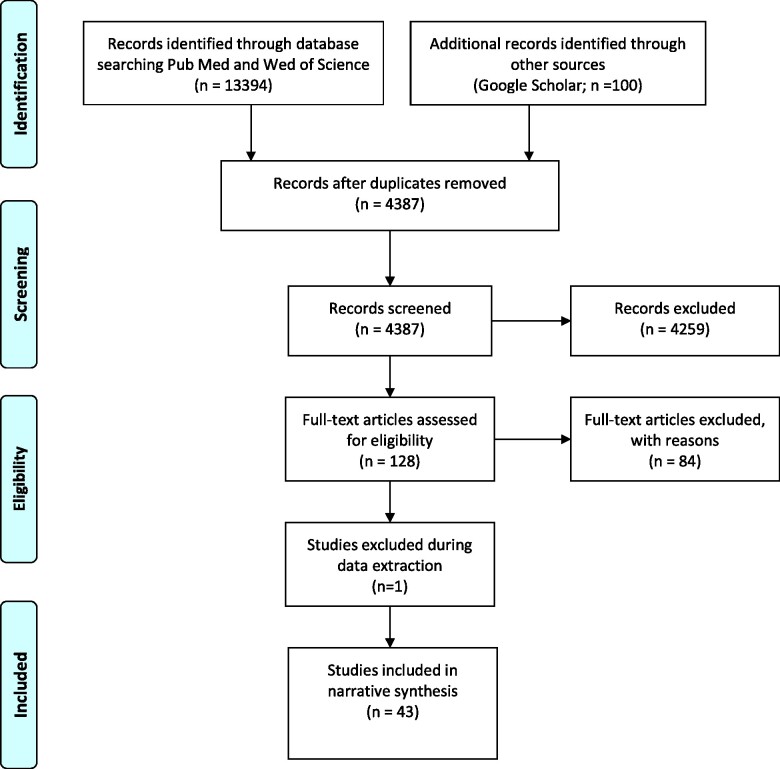
Search results and included studies.

The search results were extracted into Mendeley 1.19.4 and checked for duplicates, which were subsequently removed. As a first step, all de-duplicated titles and abstracts retrieved from the literature searches were independently screened by A1 and A2. Any doubt around whether certain studies should be included was resolved by three other researchers on the team (A3, A4 and A5). Following this screening phase, two researchers reviewed the full text of the papers to ensure that all inclusion criteria were met (A2 and A1). The selection process is summarized in Figure 1.

Articles were eligible for inclusion in this review if they:

- Were written in English, Spanish, French and Portuguese;
- Were published in peer-reviewed journals between 1990 and 2019 (conference abstracts, trial protocols, systematic reviews and non-peer-reviewed publications were excluded);
- Included a behaviour change intervention defined using the Behaviour Change Wheel (BCW) developed by Michie *et al.* (2011) and explained in further detail below (Michie *et al.*, 2011).
- Included interventions evaluated within the framework of a randomized controlled trial (RCT), interrupted time series (ITS) analyses, controlled before-after (CBA) studies, or studies that had a quasi-experimental design that would allow the establishment of causal relationships;
- Included primary and secondary outcomes that measured use of antibiotics, for example, the numbers of antibiotics prescribed by a provider, rate of antibiotic dispensing, rate of antibiotic use, etc; and
- Were undertaken in countries classified as LMIC using the World Bank’s 2019 country classification (World Bank, 2020).

### Intervention categorization

**Figure 2:**
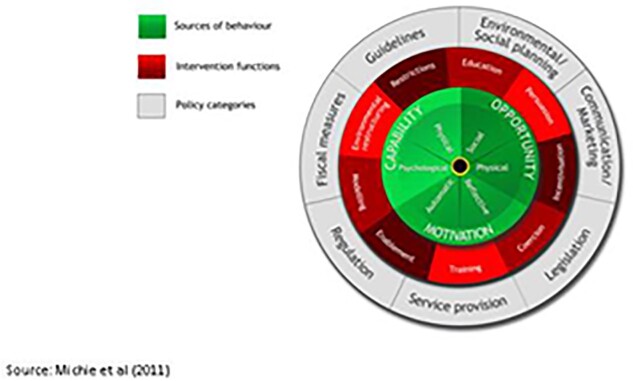
Behaviour change wheel.

The BCW is a layered framework that allows situation analysis in a step-wise manner by (1) defining the problem; (2) specifying target behaviour(s); and (3) identifying changes needed (Michie *et al.*, 2011). This can be linked to intervention functions such as training, enablement, education, etc. that might be necessary to change or shift behaviours in order to address the gaps identified. The framework then links the intervention functions to policy options that could support appropriate intervention implementation and delivery (Figure 2).

Using this framework, we categorized interventions as:

- Education: Interventions such as face-to-face lectures; small-group discussion, workshops, or seminars; refresher courses; educational outreach and visual aids that focus on imparting knowledge and developing understanding.
- Training: Interventions such as training sessions; and train-the-trainer sessions that lead to skill and capacity development.
- Modelling: Interventions where imitation acts as a motivational tool, facilitated by peer-review committees or other monitoring/regulatory committees.
- Enablement: Interventions such as feedback and audit; reminders and supervision that provide comprehensive support to trigger behaviour change by reducing barriers.
- Persuasion or coercion: Interventions that use a stimulus such as public reporting; communication/information; leaflets; posters or waiting room videos to induce action driven by the expectation of punishment or cost, or positive or negative feelings towards something.
- Incentivization: Interventions that create an expectation of a financial or non-financial reward conditional on engagement in an optimal behaviour.
- Restriction: Interventions that use the implementation of rules such as law or guideline enforcement (e.g. antibiotic stewardship programmes and antibiotic prophylaxis policies); and changes in governance structure to improve the opportunity to engage in the targeted behaviour.

### Data analysis and synthesis

Data were extracted into an Excel worksheet to capture details about the authors, country setting, type of intervention, target population, clinical or community setting and evaluation outcomes. As there was a high degree of heterogeneity in study outcomes, we conducted a narrative synthesis, whereby we collated the findings from the included studies to form a coherent description of study findings, along with differences in characteristics of the studies including context and quality (Popay *et al.*, 2006; Petticrew *et al.*, 2013; Campbell *et al.*, 2018).

### Quality appraisal

We conducted an appraisal of the quality of the included studies using the GRADE approach (Atkins *et al.*, 2004; Guyatt *et al.*, 2008), which specifies four levels of quality of evidence that range from very low to high (Table 2) (Guyatt *et al.*, 2008). Evidence from RCT studies is rated as high quality while evidence from observational studies is rated with lower quality owing to the residual confounding in this type of study design (Shünemann *et al.*, 2013).

**Table 2:** GRADE quality ratings

Quality	Meaning
Very low	True effect is probably markedly different from the estimated effect
Low	True effect might be markedly different from the estimated effect
Moderate	Authors believe that true effect is probably close to the estimated effect
High	Authors have a lot of confidence that the true effect is similar to the estimated effect

*Source:*Guyatt *et al.* (2008).

We used the five criteria recommended by Ryan and Hill (2016) to assess quality: (1) study design, (2) overall risk of bias, (3) consistency in results, (4) precision of estimates and (5) whether studies evaluate interventions relevant to the research question (Ryan and Hill, 2016). The final GRADE quality ratings were based on the application of these criteria to the included studies. The quality appraisal was led by A3 and A1.

## Results

### Search results and included studies

The search generated 4387 possible articles as shown in Figure 1. Titles, key words and abstracts were reviewed as a first check, and 4259 (97.1%) articles were excluded on this basis. The full texts for all remaining articles were then reviewed, and 85 (1.9%) were excluded. This left 43 articles (1%), which are included in this review.

### Geographical location of studies

**Figure 3:**
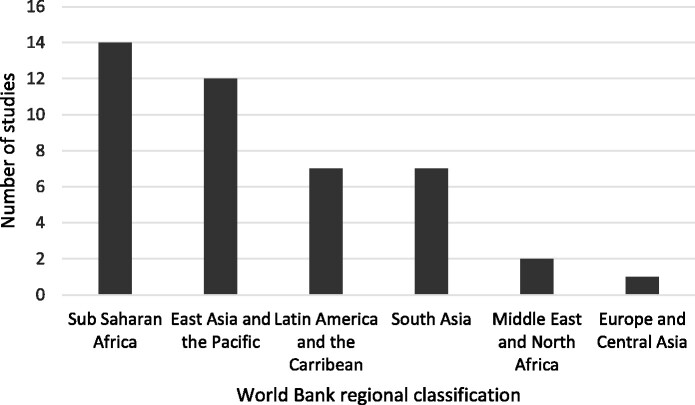
Study settings by World Bank regional classification.

The majority of the included articles evaluated interventions in one country (95.3%); only two articles (4.7%) evaluated interventions in multiple settings (Chalker *et al.*, 2005; Santa-Ana-Tellez *et al.*, 2013). Twenty-three (53.5%) were from upper-middle-income countries; followed by lower-middle-income countries (*n* = 13; 30.2%), with very few from low-income countries (*n* = 7; 16.3%). Most of the included articles were from East Asia and the Pacific region (*n* = 14; 32.6%), followed by Sub-Saharan Africa (*n* = 12; 27.9%) (Figure 3).

### Target population and study setting

Most of the articles evaluated interventions set in the public sector (*n* = 36; 83.7%). A total of 18 interventions (41.9%), were targeted at physicians (or doctors) only, six at pharmacy staff only (14%), two at nurses (4.7%), two at community health workers only (4.7%) and one at patients only (2.3%). Nine (20.9%) were targeted at multiple prescribers at health facilities such as physicians, medical officers, nursing staff but excluded all pharmacy staff, while one targeted multiple prescribers as well as pharmacy staff (2.3%). Four interventions targeted both physicians and patients (9.3%). The interventions were largely set in public health centres and clinics (Tables 3 and 4).

**Table 3:** Summary of single component behaviour change interventions

Author	Country	Intervention	Study design	Setting	Target population	Facillity ownership	Outcome(s)
Education							
Angunawela *et al.* (1991)	Sri Lanka	Two interventions arms: Written information on rational antibiotic (AB) prescribing Written information on rational AB prescribing and a seminar on AB therapy to improve AB prescribing	RCT	Outpatient; Hospital and other health facilities	Multiple prescribers	Public	Decrease in proportion of patients prescribed AB: mean difference = −7.4% in the written information group and −7.3% in written information and seminar group
Bexell *et al.* (1996)	Zambia	Continuing education seminars to improve rational drug use and patient management	RCT	Outpatient; Health centre	Multiple prescribers	Public	Mean number of drugs prescribed per patient decreased from 2.3 to 1.9 Prescribers in intervention arm improved more than in the control arm in choosing correct drug Proportion of patients prescribed AB decreased in intervention arm from 41.2% to 33.2% during, and to 34.4% after intervention. Overall improvement in recorded history taking, examination and diagnosis among intervention arm. Adequate case management improved from 18.8% before intervention, to 33.5% during intervention and to 27.8% after intervention.
Esmaily *et al.* (2010)	Iran	Continuing medical education (CME): In control arm was lecture based and used traditional teaching methods In intervention arm based on the lesson on plans from teacher training workshop, use of interactive and learner-centred teaching techniques (Q&A sessions, case studies and reports, group discussions and role play) to improve rational prescribing	RCT	Outpatient and inpatient; Primary health care	Physicians	Public	Decrease in mean number of drugs per prescription from 4.11 to 3.89 in intervention arm Decrease in mean number of injections per prescription from 0.95 to 0.80 in intervention arm.
Fairall *et al.* (2005)	South Africa	Education outreach delivered to nurses on tuberculosis case detection and primary care of respiratory illness	RCT	Outpatient; Primary health care	Nurses	Public	No difference in the prescription rates of AB commonly used for respiratory indications between groups
Ngoh *et al.* (1997)	Cameroon	Visual aids to communicate prescription drug instructions and improve compliance	RCT	Outpatient; Primary health care	Patients	Public	Intervention arm scored significantly higher than the control arm in measures of both comprehension of prescription drug instructions and compliance.
Santoso *et al.* (1996)	Indonesia	Two intervention arms: Small group face-to-face discussion Formal seminar to improve appropriate drug use	RCT	Outpatient; Primary heatlh care	Multiple prescribers	Public	Decrease in antimicrobial usage in both intervention arms: Small-group face-to-face intervention (77.4 ± 2.7% to 60.4 ± 2.9%) Formal seminar (82.3 ± 3.0% to 72.3 ± 3.6%) Small-group face-to-face intervention caused greater reduction than formal seminar
Obua *et al.* (2004)	Uganda	Educational seminar covering principles of rational drug use and National Standard Treatment Guidelines to improve prescribing practices for ARI, malaria, and non-dysenteric diarrhoea	Quasi- experimental	Outpatient; Private clinics	Physicians	Private	Decrease in AB prescribing for ARI by 23% Decrease in average cost of drugs prescribed by US$ 0.2
Akter *et al.* (2009)	Bangladesh	Education for all physicians on clinical topics to improve appropriate antimicrobial use	Quasi- experimental	Inpatient; Hospital	Physicians	Public	Increase in appropriate antimicrobial therapy for pneumonia and diarrhoea 16.4% and 56.8% respectively in intervention hospital
Training							
Meyer *et al.* (2001)	South Africa	Training‐of‐trainers course followed by effective prescribing workshops to improve prescribing practices	RCT	Outpatient; Primary health care	Nurses	Public	Lower mean number of items per prescription (1.71) compared to control arm (1.95) Lower proportion prescriptions with at least 1 AB (29.41%) compared to control arm (45.64%)
Shrestha *et al.* (2006)	Nepal	Training healthworkers to implement the WHO clinical practice guidelines to improve the management of respiratory diseases in adults	RCT	Outpatient; Primary health care	Community health workers	Public	No statistically significant difference in AB prescription Reduced the wastage cost per prescription reduced by 2.5 Nepali rupees.
Babigumira *et al.* (2017)	Malawi	Combination of classroom-based (didactic) and field-based training under supervision to improve access to essential medicines	Quasi- experimental	Outpatient; Health centre	Pharmacy staff	Public	No effect on household access to AB
Aiken *et al.* (2013)	Kenya	Development and implementation of an AB pre- and post-operative prophylaxis policy to improve prescribing behaviour	Time series	Inpatient; Hospital	Multiple prescribers	Public	Increase in adherence to AB pre-operative prophylaxis policy from 2% pre intervention to 60% in Week 1 and 98% in Week 6 after intervention. Decrease in post-operative prophylactic AB from 99% pre-intervention to 40% in Week 1 and 10% in Week 6 after intervention
Restriction							
Berild *et al.* (2008)	Russia	Development and implementation of treatment guidelines for gastrointestinal infections (GII) and respiratory tract infections (RTI) to improve AB use	Quasi- experimental	Inpatient; Hospital	Physicians	Public	In intervention ward, between baseline and follow up: Decrease in proportion of GII patients receiving AB from 94% to 73% (RD: 0.217) Decrease in the proportion of RTI patients receiving AB from 90% to 83% (RD: 0.073) Increase in proportion of cephalosporin use (RD=0.282) Compared to the control ward: Lower proportion of patients receiving cephalosporins (RD:−0.178) Lower total proportion of patients receiving AB (RD:−0.397) and furazolidone (RD: −0.125). Lower proportion of patients receiving AB and cephalosporins (RD:−0.268 and RD:−0.412 respectively), but higher for penicillin (RD: 0.099)
Chandy *et al.* (2014)	India	Implementation of policy guidelines on hospital AB use	Time series	Outpatient and inpatient; Hospital	Multiple prescribers	Public	Rising in AB use contained towards latter half of the decade, for most groups of AB. Seasonal fluctuations and variations and development of AB policy guidelines and dissemination contributed to containing use of AB
Liang *et al.* (2014)	China	Governance reform to disincentivise prescription of unnecessary and expensive medicines such as AB	Time series	Outpatient; Primary health care	Physicians	Public	Decrease in monthly proportion of patients receiving an AB injection by 9.17% Decrease in monthly proportion of patients receiving two or more AB conditional on receiving an AB decreased by 7.34% Over time, reduction in proportion of patients receiving an AB injection, average cost of AB, average number of medications and average cost of drugs per month, compared with the control series
Santa-Ana-Tellez *et al.* (2013)	Brazil and Mexico	Over-the-counter restrictions on AB consumption only to patients who present a prescription to improve AB consumption	Time series	Community; Pharmacy	Pharmacy staff	Private	In Mexico: Decrease in total AB usage from 10.5 to 7.5 DDD/TID (29.2%). Change in level of consumption of -1.17 DDD/TID Only penicillins and sulfonamides had significant changes in levels: −0.86 DDD/TID and −0.17 DDD/TID, respectively In Brazil: Increase in total AB usage from 5.7 to 8.5 DDD/TID (49.3%) Change in level of consumption of −1.35 DDD/TID. Penicillins, sulfonamides and macrolides consumption had a decrease in levels: 0.64 DDD/TID, 0.41 DDD/TID and 0.47 DDD/TID, respectively.
Persuasion							
Yang *et al.* (2014)	China	Public availability of ranking league tables of prescribing physicians and hospitals to consumers and health workers outpatient departments. To improve AB prescribing for URTIs. Ranking based on: Percentage of prescriptions requiring AB Percentage of prescriptions requiring IV injections Expenditure per prescription	RCT	Outpatient; Primary health care	Physicians	Public	Decrease in AB use for oral administration by 9 percentage points Increase in percentage of prescriptions requiring two or more AB in both intervention and control groups. Increase was 7 percentage points less in intervention arm than control arm
Liu *et al.* (2016)	China	Reports of percentage of prescriptions requiring AB, percentage of prescriptions requiring injections, and average expenditure of patients displayed at outpatient departments, sent monthly to physicians and made available to patient every 3 months to improve AB and injection prescription	RCT	Outpatient; Primary health care	Physicians	Public	Decrease in the use of combined AB (OR = 0.870). Increase in prescriptions requiring AB (OR = 1.089) or injections (OR = 1. 258)
Enablement							
Magedanz *et al.* (2012)	Brazil	Prospective audit with feedback to prescriber, with and without the presence of a pharmacist to improve AB stewardship	Quasi- experimental	Inpatient; Hospital	Physicians	Public	Reduction of 25% in antimicrobial consumption and 69% in hospital AB costs after implementation. Decrease in global consumption from 48.9 mean monthly consumption in DDD/100 patient-days during the first period, to 36.9 in the third period after implementation. Decrease in the mean monthly AB cost, from US$ 30,727.56 in the first stage of implementation to US$ 18,034.89 in the second, and US$ 9,623.73 in the last.

**Table 4:** Summary of multi-faceted behaviour change interventions

Author	Country	Intervention	Study design	Behaviour change components	Setting	Target Population	Facility ownership	Outcome(s)
Two-component interventions								
Podhipak *et al.* (1993)	Thailand	Two intervention components Face to face training workshop delivered to pharmacists; educational material sent by post to non-attendees Educational material sent by post to drug sellers to improve treatment of diarrheal diseases among these health care professionals	Quasi -experimental	Education Training	Outpatient; Pharmacy	Pharmacy staff	Private	No change in oral rehydration solution (ORS), AB and antidiarrheal drug prescription to treat watery diarrhoea among pharmacists Change in ORS usage in treatment of diarrhoea was 12% in the intervention arm compared to 8% in the control arm for drug sellers Decrease in use of AB for the treatment of dysentery in the intervention arm
Tumwikirize *et al.* (2004)	Uganda	Two intervention components: Training sessions on appropriate management of ARI in children Distribution of written materials for counter attendants in practice.	Quasi -experimental	Education Training	Outpatient; Pharmacy	Pharmacy staff	Private	Decrease in AB use for mild ARI by 3.6% and 5.8% in the intervention and control arms, respectively. Increase in AB use by 23.4% and 11.8% in the intervention and control arms, respectively Decrease in amoxicillin use by 6.8% in and 22.2% in the intervention and control arms, respectively.
Ngasala *et al.* (2008)	Tanzania	Two intervention arms: Clinical algorithm + microscopy Only clinical algorithm to improve antimalarial drug prescription and health outcomes	RCT	Education Training	Outpatient; Primary health care	Community health workers	Public	No significant difference in proportion of AB prescriptions in intervention arms.
Pérez *et al.* (2003)	Colombia	Two intervention components: Structured AB prescription form Educational campaign on correct AB prescription and graphic/poster reminders. to improve AB prescription	Time series	Education Enablement	Inpatient; Hospital	Physicians	Public	Reduction in proportion of incorrect prescriptions in aminoglycosides by 47%. Reduction in proportion of incorrect prescriptions of ceftazidime and cefotaxime by 7.3% Reduction in the proportion of incorrect prescription of surgical prophylactic AB by 20%
Awad *et al.* (2006)	Sudan	Three intervention arms: Audit and feedback Audit and feedback + seminar Audit and feedback + face-to-face educational meetings with a pharmacologist to improve AB prescribing practices of medical officers and medical assistants at health centres	RCT	Education Enablement	Outpatient; Health centre	Multiple prescribers	Public	One-month post intervention Decrease in mean number of encounters with an AB prescribed by 6.3 in audit and feedback + meeting group, 5.3 in audit and feedback + seminar group and 1.4 in audit and feedback group. Decease in the mean number of inappropriate AB prescriptions by 5.3 in the audit and feedback + meeting group, 4.4 in the audit and feedback + seminar group and 1.8 in the audit and feedback group Three months post intervention: Decrease in mean number of encounters with an AB prescribed by 7.7 in audit and feedback + meeting group, 7.1 in audit and feedback + seminar group and 2.8 in audit and feedback group. Decease in mean number of inappropriate AB prescriptions by 5.9 in audit and feedback + meeting group, 5.1 in t audit and feedback + seminar group and 1.9 in audit and feedback group
Qidwai *et al.* (2006)	Pakistan	Implementation of diarrhoea treatment as per guidelines	Quasi experimental	Education Restriction	Outpatient; Pharmacy	Pharmacy staff	Private	No statistically significant effect
Hadi *et al.* (2008)	Indonesia	Four intervention components: Development and implementation of a consensus guideline Distribution of guideline pocketbook Carrying out blood cultures free of charge Teaching sessions and refresher courses. to optimize AB usage in adults admitted with fever	Time series	Education Restriction	Inpatient; Hospital	Physicians	Public	Decrease in patients with fever in whom AB therapy is started within 24 hours after admission from 88% to 71% (effect size –17% Decrease in AB use from 99.8 to 73 DDD/100 patient‐days Increase in therapy in agreement with guideline for sepsis and dengue fever from 49% to 72% and from 58% to 88% respectively.
Zhang *et al.* (2018)	China	Training for the implementation of and application of evidence-based clinical guidelines on URTI management during consultations and monthly peer- review meetings assessing providers’ AB prescription rates Information provided to patients and caregivers on appropriate AB use verbally and via an educational leaflet along with video messages on appropriate use of AB in the waiting rooms and public areas of the hospitals	RCT	Training Persuasion	Outpatient; Hospital	Physicians and patients	Public	Reduction in mean AB prescription rate in the intervention arm of 30% compared to the control arm Cost of implementing the intervention, including training and materials was $390.65 per healthcare facility. Incremental cost of $0.03 per percentage point reduction in AB prescribing
Yip *et al.* (2014)	China	Two intervention components: Fee-for-service to a capitated budget with pay-for-performance Training on appropriate drug prescription to improve AB prescriving practices at health centres and village posts	RCT	Training Enablement	Outpatient; Primary health care	Multiple prescribers	Public	Decrease in rate of AB use of 6.6% at township level (ARR: 15 %) and 6.0% at village level (ARR: 16 %) Decrease in total expenditure per visit in intervention arm by 6% (1.04 yuan) at the village level
Atchessi *et al.* (2013)	Burkina Faso	Three intervention components: Abolition of user charges for drugs and visits to health care facilities Training on the use of the national diagnostic and therapeutic guidelines for healthcare workers Monthly supervision in every health facility to improve prescribing practices of health workers	Time series	Training Enablement	Outpatient; Primary health care	Multiple prescribers	Public	Reduction in inappropriate use of AB in malaria without comorbidity (OR = 0.48) for children aged 0-4 years
Gutiérrez *et al.* (1994)	Mexico	Two intervention components: Training workshop Peer-review of workshop output to improve physician prescription for acute diarrhoea	Quasi experimental	Training Modelling	Outpatient; Primary health care	Physicians	Public	Decrease in AB prescriptions from 35.4% to 17.7% soon after training Further decrease in AB prescriptions to 14.3% in a later period. Follow-up evaluations at 6, 12 and 18 months indicated, with slight tendency to revert to previous practices.
Chowdhury *et al.* (2007)	Bangladesh	Two intervention arms: Implementation of standard treatment guidelines (STG) for acute respiratory infections (ARI) and diarrhoea Implementation of STG for acute ARI and diarrhoea + audit to test the effectiveness of STG in influencing prescribing behaviour for ARI and diarrhoea	RCT	Restriction Enablement	Outpatient; Health complex	Physicians	Public	In the STG arm, decrease in AB prescribing in three of eight health centres. Average reduction in AB prescribing was 15.2% of encounters In the STG + audit arm, decrease in AB prescribing for ARI after intervention in six of eight health centres. Average reduction in AB prescribing was 23.7% of encounters STG audit more effective in term of reduction in AB prescribing for ARl
Rahbarimanesh *et al.* (2019)	Iran	Evaluation of patient’s electronic medical record, open communication with specialist physicians and feedback to providers on the use of AB to promote antimicrobial stewardship	Quasi experimental	Enablement Restriction	Inpatient; Hospital	Physicians	Public	Number of AB prescriptions reduced by 48% for both AB
Wattal *et al.* (2015)	India	Audit and feedback of AB prescription rates to improve prescribing rates	RCT	Enablement Persuasion	Inpatient; Hospital	Physicians	Public	No statistically significant effect
Three-component interventions								
Hoa *et al.* (2017)	Vietnam	Sequential implementation of intervention activities: Training sessions on acute respiratory infections (ARI) management and appropriate use of AB based on IMCI guidelines Training sessions on ARI case scenario management Poster distribution to improve knowledge and practices of healthcare providers for ARI	RCT	Education Training Persuasion	Outpatient and inpatient; Hospital and other health facilities	Multiple prescribers and pharmacy staff	Public/ Private	Improved AB prescribing/dispensing for mild ARI in intervention arm was 28% for compared with 3% in control arm.
Shen *et al.* (2018)	China	Information on theory and evidence-based ingredients, that included operation guidelines, public commitment, and takeaway information, along with feedback component for participating doctors on performance scores and percentages of prescribed AB to improve knowledge of rational use and the use of AB	RCT	Education Enablement Persuasion	Outpatient; Primary health care	Physicians and patients	Public	Improvement in beliefs favouring rational AB use Improvement in knowledge about side effects of AB was 74% in the intervention arm compared to 36% in the control arm Reduction in AN prescription from 89% to 62%; reduction seen consistently for respiratory or gastrointestinal tract infections.
Chalker *et al.* (2005)	Thailand and Vietnam	Sequential implementation of three 3-month interventions: Regulation enforcement visits from local emphasizing importance of prescription-only medicine legislation; Education (Hanoi: face-to-face; Bangkok: large group) Peer review (Hanoi: compulsory; Bangkok: voluntary) to improve AB dispensing practices at private pharmacies	RCT	Education Restriction Modelling	Outpatient; Pharmacy	Pharmacy staff	Private	In Hanoi, AB dispensing as requested improved significantly post-peer-review intervention: 71% vs 95%. Increase in dispensing staff asked for prescriptions, from increased 0% at baseline, to 6% post regulations, 13% post education, and 20% post peer interventions. In Bangkok, the proportion of those not asking questions and not giving advice after receiving all three interventions improved from 58% to 81%
Perez-Cuevas *et al.* (1996)	Mexico	Educational intervention, training of instructors, peer-review committee and self-appraisal of clinical performance in two institutions to improve prescribing practices for rhinopharyngitis	Quasi-experimental	Education Modelling Training	Outpatient; Primary health care	Physicians	Public	Decrease in prescription of AB from 80.7% to 62.7% in one institution and from 80.1% to 48.1% in the other. Increase in appropriate use of AB from 35.7% to 40.9% in one group and from 30% to 54.2% in the other.
Zhen *et al.* (2018)	China	Audit of prescriptions against guidelines, along with educational workships and monthly meetings with feedback	Time series	Education Restriction Enablement	Outpatient; Primary health care	Physicians	Public	No significant immediate impact on level of AB use but after implementation, rate reduced to 1.12% per month
Wahlstrom *et al.* (2003)	Lao PDR	Implementation of standard treatment guidelines and audit-feedback for improved case management of malaria, diarrhoea and pneumonia	RCT	Training Enablement Restriction	Inpatient; Hospital	Multiple prescribers	Public	Prescribers use of AB and anti‐diarrhoeal medication improved after intervention in nearly all cases
Four-component interventions								
Cundill *et al.* (2015)	Tanzania	Two intervention arms: Healthworker training + interactive workshops) Healthworker training + interactive workshops + feedback and motivational SMS messages + patient leaflets and poster to improve adherence to WHO malaria diagnosis and treatment guidelines	RCT	Education Training Enablement Persuasion	Outpatient; Primary health care	Physicans and patients	Public	No difference in prescribing of AB between arms Healthworker + patient intervention significantly reduced proportion of patients with non-malarial illness receiving an AB (ARD: 0.14). Prescribing of AB increased across all arms compared to before intervention—from 64% to 73% in control arm 67% to 75% in healthworker intervention arm and from 62% to 70% in healthworker + patient intervention arm.
Reyes-Morales *et al.* (2009)	Mexico	Three intervention components: Development evidence-based clinical guideline for appropriate ARI case management, Training of clinical tutors Educational intervention (workshops, individual clinical tutorials and round-table peer-reviewed sessions). to improve family physicians’ case management of ARI	Quasi-experimental	Education Modelling Training Restriction	Outpatient; Primary health care	Physicians	Public	Improved appropriate prescription of AB by 22.6% following peer-review sessions Improved appropriate case management by 12.6% following individual tutorials and 19.6% following peer-review sessions
Five-component interventions								
Guanche-Garcell *et al.* (2011)	Cuba	Six intervention components: Education Training Restriction (Prescription guideline development) Enablement (Prescription audit and feedback) Modelling (AB committee) Environmental restructuring measures to improve quality of AB prescription	Time series	Education Training Enablement Restriction Modelling	Outpatient and inpatient; Hospital	Physicians	Public	During the first 3 months of the intervention, inappropriate use of antimicrobials peaked, rising to 48.8% and reducing antimicrobial use to around 20%.
Wei *et al.* (2017)	China	Training on use of clinician guidelines, appropriate prescribing, monthly prescribing peer-review meetings, and caregiver education to improve AB prescription for upper respiratory tract infections (URTI) inc children	RCT	Education Training Persuasion Restriction Modelling	Outpatient; Hospital	Physicians and patients	Public	Decrease in AB prescription rate from 82% to 40% in intervention arm Absolute risk reduction in the AB prescribing rate of 29% in intervention arm No significant effect on multiple AB prescribing rate, broad-spectrum AB prescribing rate or intravenous AB prescribing rate Lower cost of AB prescription in intervention arm by ¥ 0.2

### Type of interventions

Nineteen articles evaluated interventions with a single component (44.2%) and 24 evaluated interventions with multiple components (55.8%). Interventions with a single component most commonly used education to influence behaviour (*n* = 8; 18.6%), followed by training (*n* = 4; 9.3%), restriction (*n* = 2; 4.7%) and persuasion (*n* = 2; 4.7%). Only one study used enablement as a behaviour change intervention strategy (2.3%). A summary of key characteristics of these studies is presented in Table 3.

Amongst the multi-faceted behaviour change interventions, 14 had two components (32.6%), 6 had three components (14%), 2 had four components (4.7%) and 2 had five components (4.7%) (see Table 4). Amongst these, the most common intervention component, combined with one or more components, was education (*n* = 16; 37.2%), followed by training (*n* = 12; 27.9%), enablement (*n* = 12; 27.9%) and restriction (*n* = 10; 23.3%). The most common combinations were education and training (*n* = 9; 20.9%) and education and enablement (*n* = 6; 14%). Table 4 presents a summary of key characteristics of these studies.

All interventions had varying participant follow-up periods ranging from 7 days (Ngasala *et al.*, 2008) to 5 years (Santa-Ana-Tellez *et al.*, 2013). The average follow-up period ∼12 months (361 days), and the median was 6 months (180 days).

The studies used various outcome indicators to measure change in antibiotic use, which can be categorized into three broad domains to aid synthesis. The vast majority of studies used outcome indicators that measured changes in antibiotic *prescribing* (*n* = 38; 88.4%). The interventions targeting antibiotic prescribing included: antibiotic prescription forms; face-to-face educational seminars or distribution of educational material; training workshops; and implementation of guidelines or antibiotic stewardship programmes either targeting at physicians, other prescribers such as nurses, medical officers or community health workers or pharmacy personnel. The other main outcome indicators were antibiotic *use* (Ngoh and Shepherd, 1997; Santa-Ana-Tellez *et al.*, 2013) and antibiotic *dispensing* (Tumwikirize *et al.*, 2004; Chalker *et al.*, 2005; Babigumira *et al.*, 2017). For studies focusing on antibiotic use, the target groups included patients, the community and pharmacy staff, and the behaviour change interventions implemented included education and restriction. For studies focusing on antibiotic dispensing, interventions most commonly included education, training, restriction and modelling that targeted private sector pharmacists or other pharmacy staff. Three articles had outcome measures that fell under two outcome categories. The article by Podhipak *et al.* (1993) had outcomes for both antibiotic prescription and use by patients. These interventions included education, training, restriction and enablement components targeted at health care providers, and pharmacists and drug sellers. The article by Hoa *et al.* (2017) considered outcomes of antibiotic prescription and dispensing and was targeted at health care providers and drug sellers through education, training and persuasion.

Evidence on the impact of the different interventions was mixed. Most of the interventions reported a positive impact (*n* = 30; 69.8%); 27 of these improved antibiotic prescriptions (62.8%) and the remaining three led to improvements in antibiotic use (*n* = 2; 4.7%) and antibiotics dispensed (*n* = 1; 2.3%). Eight studies (18.6%) had relatively smaller effect sizes and six studies (14%) had no statistically significant impact on antibiotic use or on antibiotic use. Amongst the single-faceted interventions (Table 3), all restriction-based interventions reported a positive impact (i.e. statistically significant improvement in antibiotic use). All but one of the eight education-based interventions reported a positive impact and the majority of the training interventions did not find a positive effect on the use of antibiotics (87.5%). Most multi-faceted interventions (Table 4) had a positive impact on the use of antibiotics (76.6%). Exceptions included an intervention combining education, training, enablement and persuasion that had a negative effect on antibiotic prescription (Cundill *et al.*, 2015) and two education and training interventions that found a positive impact on antibiotic use for some clinical conditions but not others (Podhipak *et al.*, 1993; Tumwikirize *et al.*, 2004).

### Costs and cost-effectiveness of interventions

Six articles conducted cost analyses along with the impact evaluation of the interventions (Tables 3 and 4). As a result, the methodology of the cost analyses and results were presented briefly in the articles, and thus, limited the reporting of results to descriptive outcomes. Three articles conducted cost analyses and found that behaviour change interventions reduced the costs of prescription and visits in *outpatient settings* (private practitioners, healthcare providers and community, patients and caregivers). The cost analysis by Obua (2004) nested within the evaluation of a quasi-experimental study found that an education-based intervention reduced the average cost of drugs prescribed by US$0.2. Two cost analyses were nested with an RCT framework. Yip *et al.* (2014) found that a two-component intervention based on training and enablement resulted in a decrease in total expenditure per visit by 6% at the village level, but not at larger administrative unit levels. Wei *et al.* (2017) found that a multifaceted intervention comprising education, training, persuasion, restriction and modelling components reduced the cost per antibiotic prescription by US$0.35 at 6 months after follow-up and by US$0.26 at the time of the 18 months of follow-up.

The remaining three articles that conducted cost analyses along with the impact evaluation of the interventions were done in the *inpatient setting.* These studies found that behaviour change interventions reduced costs due to a reduction in the number of prescribed antibiotics or other drugs, increases in the prescription of generic or essential drugs and reductions in the wastage of antibiotics. Shrestha *et al.* (2006) found that a training-based intervention led to a minor reduction in the cost per antibiotic prescription (<US$0.1). In their evaluation of a restriction-based intervention of different diseases, Berild *et al.* (2008) found that the average cost of antibiotics per patient decreased by 16% for patients with gastrointestinal infections and pneumonia patients but increased by 38% for patients with respiratory tract infections. Magedanz *et al.* (2012) estimated that an enablement-based intervention led to an overall decrease in the mean monthly cost of antibiotics from ∼US$31 000 at baseline to ∼US$10 000 post-intervention.

Only one article presented results from a full economic evaluation conducted alongside the evaluation of an RCT, comparing costs and consequences of a behaviour change intervention as a stand-alone analysis (Zhang *et al.*, 2018). Zhang *et al.* (2018) found that a multifaceted training and persuasion programme when embedded into routine practice had an incremental cost of $0.03 per percentage point reduction in antibiotic prescribing and was highly cost‐effective from the provider perspective compared to the alternative scenario i.e. with no training and implementation of guidelines. The brevity in reporting costing methodology and results in six of the seven papers included in the review, and heterogeneity in the study design, scale and cost outcomes precludes us from making robust comparisons between studies.

### Type of study and quality appraisal

The majority of the included articles used an RCT design (*n* = 22), which is considered high-quality as per the GRADE criteria (Table 2). Of the remaining 21 studies, 9 had an ITS study design and 12 had a quasi-experimental design (Tables 3 and 4). These are classified as a lower quality based on the GRADE criteria.

## Discussion

Globally, several behaviour change interventions have been implemented, along with considerable investment to combat ABR in the last three decades. To date, most of the evidence on effectiveness has been from high-income settings and previous syntheses of the literature support this (Arnold and Straus, 2009; Charani *et al.*, 2011; Davey *et al.*, 2013, 2017; Cross *et al.*, 2017). Only one review focused solely on interventions implemented in LMICs (Wilkinson *et al.*, 2018) but did not explicitly focus on behaviour change or include demand-side interventions. These reviews did not include any evidence on the costs and cost-effectiveness of interventions to improve antibiotic use. To address this evidence gap, we synthesized and appraised 43 papers evaluating the effectiveness and/or cost-effectiveness of behaviour change interventions to improve the use of antibiotics in LMICs.

Overall, our findings indicate that multi-faceted interventions were more effective in improving antibiotic use than single-faceted behaviour change interventions; however, the degree of improvement in most interventions was <20%. This finding is consistent with previous systematic reviews from high-income countries (Arnold and Straus, 2009; Charani *et al.*, 2011; Davey *et al.*, 2017; Cross *et al.*, 2017) and with the review by Wilkinson *et al.* (2018). Interventions based on education, restriction and training, either as a stand-alone intervention or as part of a multi-faceted intervention showed a positive impact on the use of antibiotics. Reflecting on these intervention functions and the policies that they link to in the BCW framework (Figure 2), it is likely policies that focus on developing and implementing guidelines, along with appropriate and context-relevant environmental and social planning can improve the use of antibiotics. The most common intervention type was education, closely followed by training. However, unlike education interventions, training interventions were more likely to succeed when combined with an education, restriction or enablement intervention component. Some have argued that this occurs because the motivational and capability development effects of education can bolster the impact of the training component (Michie *et al.*, 2011), and thus, policies that focus on environmental/social planning alone may be less effective in improving the use of antibiotics.

Accommodating all studies evaluating behaviour change interventions to improve antibiotic use in LMIC, meant that comparisons of results were difficult due to variation in settings, target populations, study designs and outcome measures. Nonetheless, some methodological implications are noteworthy. We included studies with experimental, quasi-experimental and time-series designs. As these are analytic study designs (Peinemann *et al.*, 2013), they allow us to infer causality of certain behaviour change strategies on the use of antibiotics to some degree. However, more than half of the included studies were classified as low-quality by the GRADE checklist as they did not employ an RCT design. On the one hand, non-RCT studies may not be able to account for confounders, consider adequate dose–response and/or the fact that all plausible biases could have an impact on the treatment effect (Goldet and Howick, 2013; Ryan and Hill, 2016), thereby reducing confidence in the accuracy of results. On the other hand, RCTs may provide exaggerated estimates of effect, may not be able to wholly eliminate bias (Jadad and Rennie, 1998), or have generalizable results (Hariton and Locascio, 2018). Strategies to curb ABR much tackle multi-dimensional behaviours in clinical care and community settings, highlighting the need for complex interventions. Limiting the evaluation design to RCTs may deter the implementation and assessment of public health interventions, especially when implemented at a large scale or in multiple sites (Sanson-Fisher *et al.*, 2007).

In addition, we identified a large variation in how outcomes were measured and reported across different domains of antibiotic use i.e. antibiotic prescription, dispensing and consumption. Even within the same domain, different indicators or metrics were used. This prohibited a meta-analysis and subgroup analysis (Dwan, 2011). Some studies also reported multiple outcomes, which leads to a consideration of which outcome(s) should be considered when synthesizing evidence on the effectiveness and posing challenges to a straightforward interpretation of the evidence (Mayo-Wilson *et al.*, 2017). Further, few evaluations measured the impact of behaviour change interventions over >12 months. While the majority of the studies had a short follow-up period (for example, Podiphak, 1993; Meyer 2001; Liang, 2014; Yang, 2014; Babigumira, 2017; Hoa, 2017; Wei, 2017; Zhang, 2018), some did consider the importance of longer-term benefit assessment by having an evaluation period of 18 months after the intervention (Gutierrez *et al.*, 1994; Pérez-Cuevas *et al.*, 1996). Some also recognized the importance of the benefits of program continuation beyond the project period for the yield of long-term benefit (Bexell, 1996; Chandy, 2014). It is widely recognized that the time-treatment interaction can lead to impacts that can either dissipate over time, or change direction (Cooper, 1989). Caution is therefore required when interpreting the results of current evaluations of behaviour change interventions that are based on short-term time horizons.

An evaluation of the sustainability of the interventions was challenged by the fact that the duration of the interventions and the assessment period within which the impact of the intervention was measured varied widely among studies. Some of the studies included in this review, however, discuss the importance of measuring the longer-term effect of the interventions, with one study (Berild *et al.*, 2008) highlighting that the impact of the intervention level went back to the pre-intervention level after a 1-year period of assessment. Future studies might also consider how other factors such as staff turnover, health system settings and social and cultural context could have an impact on the sustainability of a positive study outcome.

Our review identified several key gaps in the existing evidence base. First, most included articles evaluated interventions implemented in middle-income countries and only a handful were set in low-income countries. Those that were implemented in low-income countries provided mixed evidence on the kinds of behaviour change interventions that can have a positive impact on antibiotic use. This presents a significant gap in the evidence base for these countries, many of which have been tasked with developing a national action plans to curb ABR (World Health Organization (WHO), 2015b). The lack of adequate evidence on how antibiotics and other essential medicines are used, different patterns of ABR amongst the population, and how these change over time, limits the development of effective policy strategies such as regulation, legislation, changes to service provision or implementation of fiscal measures to improve antibiotic use. This may be overcome by developing effective and reliable ABR surveillance systems that can integrate surveillance of ABR in human, animal and food-borne pathogens to provide comprehensive and dynamic situation analyses; information on overall mortality and morbidity; and capture the extent of the economic and social impacts of ABR (World Health Organization (WHO), 2014; Holloway *et al.*, 2017). The importance of such surveillance and research has been globally recognized in the WHO’s global action plan to tackle antibacterial resistance as a way of generating knowledge and translation into policy action (World Health Organization (WHO), 2015a). However, it may be less feasible to do so in countries where health systems are too constrained to allow appropriate allocation of resources to improve the use of antibiotics.

Second, the majority of articles evaluated interventions targeting the behaviour of health care providers and only a few focused on patients and the wider community. The inappropriate use of antibiotics is highly influenced by human behaviour at many levels of society. Interventions targeting patients and the wider community, potentially using communication and environmental/social planning policies (Charani *et al.*, 2011) are required to improve the use of antibiotics (Ayukekbong *et al.*, 2017) and leaving out these key agents could hinder efforts to tackle ABR (Radyowijati and Haak, 2003; Holloway, 2011; Suy *et al.*, 2019).

Third, only five studies in this review targeted healthcare providers outside the public sector. Although inappropriate use of antibiotics, owing at least partially to perceived demand from patients (Kotwani *et al.*, 2010; Om *et al.*, 2016), is known to occur at public and private health facilities, the relative lack of studies focusing on the private sector presents a considerable challenge to tackling ABR in LMICs where private drug sellers (including community pharmacies, drug shops and general stores) are the first primary contact point for outpatient services such as consultations, diagnoses, drug prescription and dispensing (Goel *et al.*, 1996; Kwena *et al.*, 2008). Antibiotics are typically purchased from these providers without a prescription and/or dispensed by personnel without adequate training (Lansang *et al.*, 1990; Ayukekbong *et al.*, 2017). The severity of this is illustrated through a recent systematic review, which found 62% of antibiotics were dispensed without a prescription in community pharmacies globally (Auta *et al.*, 2019).

Fourth, the Medical Research Council’s framework for the evaluation of complex interventions recommends that in addition to looking at the effect of the intervention, studies should also conduct economic and process evaluations to support research translation (Craig *et al.*, 2008). Process evaluations provide key evidence on the fidelity and the quality of implementation of interventions, clarification of causal pathways and means to identify any context-related factors that can lead to variations in outcomes (Craig *et al.*, 2008). No studies in this review included a process evaluation. Without process evaluation results, policymakers lack adequate information on the barriers to or facilitators of success of behaviour change interventions to improve antibiotic use in a specific context; thus, reducing the likelihood of replication or uptake in another context (Moore *et al.*, 2015).

Seven articles presented results from an economic evaluation of an intervention, of which six presented the results of a cost analysis conducted along with the main intervention evaluation. Only one article presented results from a full economic evaluation as a stand-alone analysis. The brief and descriptive nature of reporting economic evaluation methods and outcomes in the majority of studies and the heterogeneity in the scope of the economic evaluation, perspectives, scale and outcomes posed a challenge in making robust comparisons between interventions. While indicative of the relative value of investments in interventions that have the potential to address the public health problem that ABR poses, this evidence gap limits a decision-maker’s ability to compare between different programmes. Thus, evaluations of interventions should ideally be accompanied by a full economic evaluation that adheres to established guidelines and reports on the cost-effectiveness of the intervention for the trial duration, and for a long-term horizon would be beneficial for decision-makers. This would provide more rigorous evidence on the costs and benefits of such interventions as well as on the budget impact or affordability to aid decisions about the efficiency of intervention delivery, priority setting, financial planning and management and the formulation of resource requirements and budgets (Vassall *et al.*, 2017). Thus, in our view, rigorous impact evaluations accompanied by process and economic evaluations that adhere to a published study protocol and provide transparency in the form of a declaration of conflict of interests amongst evaluators would allow policymakers to gauge whether a clinically effective intervention may be scalable and/or replicable within the same context or another, and aid a priority setting exercise that could lead to maximizing population health in the presence of health systems constraints.

Our review did not include any grey literature. This presents a possibility that we have excluded evidence on successful interventions implemented by government and/or non-government institutions. While including grey literature could potentially increase the comprehensiveness of the synthesized evidence, it also presents risks as studies may not always follow gold-standard or recommended guidelines for evaluation or may not be peer-reviewed (Adams *et al.*, 2016).

Our research question is specific to whether behaviour change interventions can reduce antibiotic prescription, and therefore, our search terms are tailored to this objective. We included all studies that had antibiotic use and prescription as a primary, secondary or intermediate impact. We believe that the risk that papers where an improvement in antibiotic use through behaviour change interventions was a spill-over effect may have been captured but some publications may have been excluded because of titles, keywords and abstracts that did not explicitly match our research objective or search criteria.

Antibiotics remain a powerful and effective treatment for bacterial infections, but inappropriate use can pose a threat to health and well-being. Our review found that there are several effective behaviour strategies that can be implemented to improve antibiotic use in LMICs. However, the evidence base is heavily skewed towards healthcare providers with far less attention having been paid to improving antibiotic use amongst patients and the general public. Moreover, given the importance of private drug sellers in the provision of antibiotics in LMICs, it was surprising to see so few studies targeting these providers. From a design perspective, future studies in this field would also benefit from including longer time horizons for follow-up or more follow-up points to understand how the impact of interventions is sustained over time; process evaluations to understand the facilitators of and barriers to behaviour change; and full economic evaluations. Addressing these gaps will help to gain a clearer understanding of effective, sustainable and scalable approaches to tackle ABR, and in the long-term improve the health outcomes of individuals, and reduce resource burdens on household, families and health systems (Founou *et al.*, 2017).

*Conflict of interest statement*. The authors have no conflicting interests.

*Ethical approval.* No ethical approval was required for this study.
