# Correction to: Improving antibiotic use through behaviour change: a systematic review of interventions evaluated in low- and middle-income countries

**DOI:** 10.1093/heapol/czae107

**Published:** 2024-11-22

This is a correction to: Carla Cuevas, Neha Batura, Luh Putu Lila Wulandari, Mishal Khan, Virginia Wiseman, Improving antibiotic use through behaviour change: a systematic review of interventions evaluated in low- and middle-income countries, *Health Policy and Planning*, Volume 36, Issue 5, June 2021, Pages 754–773, https://doi.org/10.1093/heapol/czab021.

In the published version of this manuscript, some of the papers listed in Table 3 and Table 4 were inadvertently omitted from the reference list. The missing references are provided below, along with corrections to the citation of Hadiyono JE, Suryawati S, Danu SS, Santoso B. and Yang, L. et al. in Table 3.

These details have been corrected only in this correction notice to preserve the version of record.

Missing references from Table 3:

- Angunawela II, Diwan VK, Tomson G. 1991. Experimental evaluation of the effects of drug information on antibiotic prescribing: a study in outpatient care in an area of Sri Lanka. *Int J Epidemiol* 20: 558–564.
- Bexell A, Lwando E, von Hofsten B, Tembo S, Eriksson B, Diwan VK. Improving drug use through continuing education: a randomized controlled trial in Zambia. Journal of Clinical Epidemiology. 1 March 1996;49(3):355–7.
- Fairall LR, Zwarenstein M, Bateman ED, Bachmann M, Lombard C, Majara BP, Joubert G, English RG, Bheekie A, van Rensburg D, Mayers P. Effect of educational outreach to nurses on tuberculosis case detection and primary care of respiratory illness: pragmatic cluster randomized controlled trial. Bmj. 29 September 2005;331(7519):750–4.
- Ngoh LN, Shepherd MD. Design, development, and evaluation of visual aids for communicating prescription drug instructions to nonliterate patients in rural Cameroon. Patient education and counselling. 1 July 1997;31(3):245–61.
- Hadiyono JE, Suryawati S, Danu SS, Santoso B. Interactional group discussion: results of a controlled trial using a behavioural intervention to reduce the use of injections in public health facilities. Social science & medicine. 1 April 1996;42(8):1177–83. This should be referred to as Hadiyono et al. (1996) in the table.
- Obua C, Ogwal-Okeng JW, Waako P, Aupont O, Ross-Degnan D. Impact of an educational intervention to improve prescribing by private physicians in Uganda. East Afr Med J. 2004 Feb; Suppl:S17-24. PMID: 15125112.
- Akter, S. F. U. et al. (2009) ‘Impact of a training intervention on use of antimicrobials in teaching hospitals’, *JOURNAL OF INFECTION IN DEVELOPING COUNTRIES*, 3(6), pp. 447–451.
- Meyer, J. C., Summers, R. S. and Möller, H. (2001) ‘Randomized, controlled trial of prescribing training in a South African Province’, *Medical Education*, 35(9), pp. 833–840. doi: 10.1046/j.1365-2923.2001.01000.x.
- Shrestha, N. et al. (2006) ‘Practical Approach to Lung Health in Nepal: Better prescribing and reduction of cost’, *Tropical Medicine and International Health*. England, 11(5), pp. 765–772. doi: 10.1111/j.1365-3156.2006.01599.x.
- Chandy, S. J. et al. (2014) ‘The impact of policy guidelines on hospital antibiotic use over a decade: a segmented time series analysis.’, *PloS one*. United States, 9(3), p. e92206. doi: 10.1371/journal.pone.0092206.
- Yang, L. et al. (2014) ‘Public reporting improves antibiotic prescribing for upper respiratory tract infections in primary care: a matched-pair cluster-randomized trial in China’, *HEALTH RESEARCH POLICY AND SYSTEMS*, 12. doi: 10.1186/1478-4505-12-61. Sometimes this is referred to as 2004 in the text but it should be 2014 all through.
- Magedanz, L., Silliprandi, E. M. and dos Santos, R. P. (2012) ‘Impact of the pharmacist on a multidisciplinary team in an antimicrobial stewardship program: a quasi-experimental study.’, *International journal of clinical pharmacy*. Netherlands, 34(2), pp. 290–294. doi: 10.1007/s11096-012-9621-7.

Missing references from Table 4:

- Pérez, A. et al. (2003) ‘An interrupted time series analysis of parenteral antibiotic use in Colombia’, *Journal of Clinical Epidemiology*, 56(10), pp. 1013–1020. doi: 10.1016/S0895-4356(03)00163-X.
- Awad, A. I., Eltayeb, I. B. and Baraka, O. Z. (2006) ‘Changing antibiotics prescribing practices in health centers of Khartoum State, Sudan.’, *European journal of clinical pharmacology*. Germany, 62(2), pp. 135–142. doi: 10.1007/s00228-005-0089-4.
- Qidwai, W. et al. (2006) ‘Private drug sellers’ education in improving prescribing practices’, *Journal of the College of Physicians and Surgeons Pakistan*, 16(12), pp. 743–746. doi: 12.2006/jcpsp.743746.
- Hadi, U. et al. (2008) ‘Optimizing antibiotic usage in adults admitted with fever by a multifaceted intervention in an Indonesian governmental hospital’, *Tropical Medicine and International Health*, 13(7), pp. 888–899. doi: 10.1111/j.1365-3156.2008.02080.x.
- Atchessi, N., Ridde, V. and Haddad, S. (2013) ‘Combining user fees exemption with training and supervision helps to maintain the quality of drug prescriptions in Burkina Faso’, *HEALTH POLICY AND PLANNING*, 28(6), pp. 606–615. doi: 10.1093/heapol/czs100.
- Gutierrez, G. et al. (1994) ‘Changing physician prescribing patterns: evaluation of an educational strategy for acute diarrhea in Mexico City.’, *Medical care*. United States, 32(5), pp. 436–446.
- Chowdhury, A. K. et al. (2007) ‘Effect of standard treatment guidelines with or without prescription audit on prescribing for Acute Respiratory Tract Infection (ARI) and diarrhoea in some Thana Health Complexes (THCs) of Bangladesh’, *Bangladesh Medical Research Council Bulletin*, 33(1), pp. 21–30.
- Rahbarimanesh, A. et al. (2019) ‘Antimicrobial stewardship program (ASP): an effective implementing technique for the therapy efficiency of meropenem and vancomycin antibiotics in Iranian pediatric patients.’, *Annals of clinical microbiology and antimicrobials*. England, 18(1), p. 6. doi: 10.1186/s12941-019-0305-1.
- Wattal, C. et al. (2015) ‘Impact of informational feedback to clinicians on antibiotic-prescribing rates in a tertiary care hospital in Delhi’, *Indian Journal of Medical Microbiology*, 33(2), p. 255. doi: 10.4103/0255-0857.153582.
- Hoa, N. Q. et al. (2017) ‘Antibiotic prescribing and dispensing for acute respiratory infections in children: effectiveness of a multi-faceted intervention for health-care providers in Vietnam’, *GLOBAL HEALTH ACTION*, 10. doi: 10.1080/16549716.2017.1327638.
- Shen, X. et al. (2018) ‘Web-Based Just-in-Time Information and Feedback on Antibiotic Use for Village Doctors in Rural Anhui, China: Randomized Controlled Trial:, 20, pp. 1–13. doi: 10.2196/jmir.8922.
- Perez-Cuevas, R. et al. (1996) ‘Improving physician prescribing patterns to treat rhinopharyngitis. Intervention strategies in two health systems of Mexico.’, *Social science & medicine (1982)*. England, 42(8), pp. 1185–1194.
- Zhen, L., Jin, C. and Xu, H.-N. (2018) ‘The impact of prescriptions audit and feedback for antibiotic use in rural clinics: interrupted time series with segmented regression analysis.’, *BMC health services research*. England, 18(1), p. 777. doi: 10.1186/s12913-018-3602-z.
- Reyes-Morales, H. et al. (2009) ‘A multifaceted education intervention for improving family physicians’ case management.’, *Family medicine*. United States, 41(4), pp. 277–284.
- Guanche Garcell, H. et al. (2011) ‘[Impact of a quality control program on antibiotic prescription in a hospital in Havana, Cuba].’, *Revista panamericana de salud publica = Pan American journal of public health*. United States, 30(6), pp. 598–602.
- Wei, X. et al. (2017) ‘Effect of a training and educational intervention for physicians and caregivers on antibiotic prescribing for upper respiratory tract infections in children at primary care facilities in rural China: a cluster-randomised controlled trial.’, *The Lancet. Global health*. England, 5(12), pp. e1258–e1267. doi: 10.1016/S2214-109X(17)30383-2.

